# Hydrogen‐Enriched Hyaluronic Acid Dressing Ameliorates Diabetic Foot Ulcer via Promoting Mitophagy

**DOI:** 10.1111/1753-0407.70209

**Published:** 2026-03-29

**Authors:** Ziyu Xu, Xinyu Cui, Houbin Chu, Hao Wan, Yunbo Xie, Ying Wang, Qingbin Ni, Xiaolin Ding, Guohua Song

**Affiliations:** ^1^ The Second Affiliated Hospital of Shandong First Medical University Taian China; ^2^ School of Basic Medical Sciences Shandong First Medical University & Shandong Academy of Medical Sciences Jinan China; ^3^ Zaozhuang Municipal Hospital Zaozhuang China; ^4^ Taian Central Hospital Taian China

**Keywords:** diabetic foot ulcer, hydrogen‐enriched hyaluronic acid dressing, mitophagy, SIRT3

## Abstract

**Objective:**

Diabetic foot ulcer (DFU) is one of the most common chronic complications of diabetes. This study developed a hydrogen‐enriched hyaluronic acid (HA) dressing and aimed to explore its therapeutic effects and mechanisms in DFU treatment.

**Methods:**

A combination of vacuum‐assisted closure (VSD) and hydrogen‐rich saline was used to treat DFU patients and assess the clinical outcomes of wound repair. A rat model of DFU was established, and treatment with hydrogen‐enriched HA dressing. Subsequently, the protective effects of the dressing were evaluated, including histological studies, the expression of inflammatory factors and angiogenesis markers. Western blot was used to analyze the expression levels of mitophagy‐related proteins. In vitro, the role of HA and hydrogen on cell mitochondrial damage, apoptosis, migration, and markers associated with mitophagy pathways in human foreskin fibroblast‐1 (HFF‐1) was assessed.

**Results:**

VSD combined with hydrogen‐rich saline significantly enhanced wound healing in patients, while reducing inflammation and oxidative damage. In vivo studies showed that the dressing promoted wound healing, increased collagen deposition, reduced inflammatory cytokines, and enhanced neovascularization. In vitro studies, high glucose induced cell morphological damage and oxidative stress, disrupted mitochondrial membrane potential, leading to apoptosis and attenuating cell migration. However, both HA and hydrogen significantly induced SIRT3 expression and activated the downstream FOXO3A/PINK1‐PARKIN signaling pathway, promoting mitochondrial autophagy and reducing cell apoptosis. Furthermore, the SIRT3/SOD2 pathway was also activated, decreasing reactive oxygen species (ROS) production and enhancing migration.

**Conclusion:**

This study confirmed that the hydrogen‐enriched HA dressing has the potential to enhance diabetic wound repair.

## Highlights

- The hydrogen‐enriched hyaluronic acid dressing exerts healing benefits for diabetic foot ulcer (DFU) in vivo and in vitro.
- The dressing affects the apoptosis and migration capacity of skin fibroblasts via promoting mitophagy.
- Treatment with the dressing reverses SIRT3‐mediated mitophagy and oxidative stress in DFU.

## Graphical abstract

The combination of VSD and hydrogen‐rich saline is used to treat diabetic foot ulcers. In vitro and in vivo studies reveal that hydrogen‐enrich hyaluronic acid dressings enhance skin fibroblast repair ability by regulating mitophagy via the SIRT3 and its downstream signaling pathways, ultimately accelerating wound healing in diabetic foot ulcers.

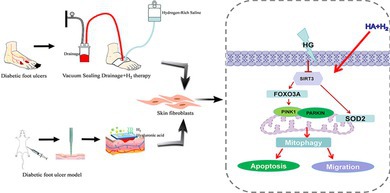

## 1 Introduction

Diabetes is a common disease that affects approximately 537 million people worldwide, which is predicted to increase to 783 million by 2045 [1, 2]. For patients with diabetes, the lifetime risk of developing foot ulcers is estimated to be as high as 34%. The number of patients with DFU is rapidly increasing, so effective strategies for treating DFU are urgently needed [3]. The presence of the wound leads to impaired functional status, infection and inflammation, lower limb amputation, and even death. Therefore, the treatment of DFU aims to promote healing, prevent infection, and reduce the risk of complications such as amputation.

Wound healing is a dynamic, complex, and sequential biological process involving the coordinated involvement of various cells, growth factors, and extracellular matrix (ECM) [4]. The wound healing process usually consists of four stages: hemostasis, inflammation, proliferation, and remodeling [5]. However, in DFU patients, elevated blood glucose exacerbates the inflammatory response, prolonging the inflammatory phase and increasing the production of pro‐inflammatory cytokines. Furthermore, hyperglycemia induces oxidative stress through the overproduction of reactive oxygen species (ROS), leading to cellular damage and suppression of fibroblast and endothelial cell functions, thereby hindering tissue repair [6]. Hyperglycemic conditions also downregulate the expression of angiogenic factors and limit neovascularization. Moreover, elevated glucose levels disrupt fibroblast proliferation, migration, and collagen synthesis, further delaying wound closure [7]. The complexity of the mechanism underlying wound healing is the major difficulty for DFU treatment.

Recent common therapies targeting wound repair mainly include newer wound dressings, such as hydrocolloids, hydrogels and foam dressings. Several pilot researches have also identified that hydrogen has shown anti‐inflammatory and antioxidant properties in wound healing [8]. These properties make hydrogen potentially valuable in the treatment of skin damage. Existing studies have shown that the apoptotic index of radiation‐damaged rat cells was significantly reduced by inhalation of hydrogen [9]. Furthermore, exposure to hydrogen‐rich culture medium can protect human fibroblasts from oxidative damage induced by high glucose [10]. However, with traditional hydrogen inhalation and hydrogen‐rich water rinses, the hydrogen molecules may escape quickly, resulting in a short residence time, which limits the therapeutic effect of hydrogen. Considering hyaluronic acid (HA) is the major component of both hydrogels and the ECM, offering ideal properties such as biocompatibility and hydrophilicity [11]. It has been found to be a promising candidate to promote wound healing by stimulating the proliferation of fibroblast, keratinocyte migration, and remodeling of the ECM [12]. Moreover, several studies have performed systematic reviews and meta‐analyses to evaluate the efficacy of HA and its derivatives in promoting wound healing in DFU [13]. In our research, we use HA to encapsulate hydrogen, it harnesses the therapeutic benefits of HA while achieving a more prolonged release of hydrogen, enhancing the local therapeutic effect. Meanwhile, in clinical settings, a combination of vacuum sealing drainage (VSD) and hydrogen‐rich water is employed to simulate the therapeutic approach of HA‐encapsulated hydrogen deliver.

Impaired mitophagy is increasingly recognized to delay wound healing [14]. Mitophagy plays a crucial role in regulating cell apoptosis and migration. It can prevent apoptotic signaling and regulate energy production by selectively removing dysfunctional mitochondria, thus influencing cell survival [15]. Additionally, mitophagy modulates the mitochondrial dynamics and intracellular signaling pathways that are crucial for cell migration, impacting processes such as wound healing and metastasis [16]. SIRT3, a member of the sirtuin family of silent information regulators, is the most important deacetylase in the mitochondria [17]. Notably, mitochondrial sirtuins have been highlighted to be closely associated with skin physiology and pathological repair, among which SIRT3 stands out for its regulatory dominance in mitophagy [18]. Some studies have also shown that SIRT3 mediates mitophagy through the FOXO3A/PINK1‐PARKIN pathway, exerting a protective effect on high glucose‐induced retinal pigment epithelial cells [19], it also has been verified to alleviate diabetic neurological damage by activating mitophagy via the FoxO3a‐PINK1‐Parkin signaling pathway, further confirming its pivotal role in diabetic‐related pathological processes through mitophagy regulation [20]. Furthermore, the expression of SIRT3 is significantly reduced in the skin wounds of diabetic patients [21]. Moreover, overexpression of SIRT3 can alleviate mitochondrial oxidative stress and reduce vascular inflammation, thereby protecting vascular function [22]. However, the potential role of hydrogen in DFU healing through SIRT3‐mediated mitophagy is still not fully understood.

Based on the above, this study aims to develop a hydrogen‐enriched HA dressing and investigate whether its therapeutic mechanism involves the regulation of mitophagy through SIRT3 and its downstream pathways, thereby facilitating the healing of DFU.

## 2 Materials and Methods

### 2.1 Ethical Practices

This study included 60 cases of DFU who meet the criteria, from the Department of Foot and Ankle Surgeryrom, Second Affiliated Hospital of Shandong First Medical University, hospitalized between December 2021 and October 2023. Participants were randomly assigned into two groups (30 cases each) using the SPSS 27.0 random number table by an individual who was not involved in the research. The groups were VSD group (normal saline) and VSD + H_2_ group (hydrogen‐rich saline). The allocation was concealed in sequentially numbered envelopes to ensure hidden randomization. The intervention was implemented by unblinded personnel, while the participants remained blinded. To minimize measurement bias, the statisticians were blinded to the study protocol. The study was approved by the Ethics Committee of the Second Affiliated Hospital of Shandong First Medical University on March 17, 2023 and registered with the China Clinical Trial Registration Center (ChiCTR2300069537). The inclusion criteria and descriptive characteristics of the participants are presented in Tables S1 and S2.

### 2.2 Treatment Plan

#### 2.2.1 VSD Implementation

Both groups of patients received routine basic treatment, followed by VSD therapy. Under epidural or nerve block anesthesia, debridement was performed with strict adherence to sterile techniques, after which the wound was soaked in diluted iodine for 10–15 min and rinsed. A negative‐pressure wound dressing was applied and sutured in place based on the ulcer's location and blood supply, and the surrounding skin was disinfected. A semipermeable membrane was used to seal the wound, extending 3–5 cm beyond the normal skin margin, and was connected to a negative pressure suction device set to −150 to −225 mmHg. The dressing was monitored for tightness and air leaks, and the drainage tube was kept unobstructed to ensure proper functioning. During the treatment process, three patients experienced device air leakage, which was promptly addressed by adding a biological membrane for coverage. After 7 days, the VSD device was removed, and relevant healing indicators were assessed.

#### 2.2.2 Preparation of Hydrogen‐Rich Saline

Hydrogen‐rich saline was prepared according to the method described by Professor Xuejun Sun [23]: A 500 mL bag of normal saline was used. Two hundred and fifty milliliter of saline and excess air were removed with a sterile syringe. Pure hydrogen was injected into the saline bag using a hydrogen generator, repeating the process two to three times. The solution was then pressurized (0.4 MPa) for 2 h to dissolve the gas and stored at 4°C in the dark. The entire process was performed under sterile conditions. The hydrogen content reached 0.6 mmol/L, as measured using the hydrogen microelectrode method [24] at the Second Affiliated Hospital of Shandong First Medical University.

#### 2.2.3 Wound Irrigation

*VSD + H*_*2*_ *Group (hydrogen‐rich saline)*: Hydrogen‐rich saline (20–50 mL) was prepared and injected through the irrigation tube. Once the dressing and drainage tube were clear, the negative pressure was turned off. The solution filled the dressing or drained out, and then the injection stopped. The solution was left for 20 min for the reaction, then negative pressure was reapplied to aspirate the fluid. This procedure was performed twice daily until the VSD device was removed on Day 7.

*VSD control group (normal saline group)*: 20–50 mL of 0.9% sodium chloride was used as the irrigation solution, following the same procedure as the VSD + H_2_ group.

### 2.3 Clinical Indicators

#### 2.3.1 Efficacy Outcome Measurement

Photographs of the wound were taken before and after treatment, and the wound area was measured using ImageJ software (in cases where the ulcerated wound is distributed across different planes, separate photographs should be taken for each plane, and the area of each plane should be measured individually. The total ulcer area is obtained by summing the areas of all planes). Based on the measured wound area, the wound healing rate and speed were subsequently calculated.

#### 2.3.2 Serum Inflammatory Markers

Fasting venous blood was collected before and after treatment and placed in blood collection tubes. The samples were promptly sent to the Department of Medical Laboratory at the Second Affiliated Hospital of Shandong First Medical University for analysis of C‐reactive protein (CRP), tumor necrosis factor‐α (TNF‐α), and interleukin‐6 (IL‐6). All blood samples were analyzed in the same laboratory, and the results were subjected to statistical analysis.

#### 2.3.3 Oxidative Stress Markers

Tissue samples were collected from the center of the wound site before and after treatment, and a 10% skin tissue homogenate was prepared. The levels of oxidative stress markers in the tissue were determined by a SpectraMax microplate reader (Molecular Devices, USA) using MDA and SOD assay kits (Nanjing Jiancheng Bioengineering Institute, China) according to the manufacturer's instructions.

### 2.4 Animal

Sprague Dawley (SD) rats (male, aged 8 weeks) were purchased from Jinan Pengyue Experimental Animal Breeding Co. Ltd. (Shandong, China) and bred in the laboratory at Shandong First Medical University. Animal studies were approved by the Institutional Animal Research Committee of Shandong First Medical University and confirmed with the US Public Health Service Policy on Humane Care and Use of Laboratory Animals (Ethical No. W202504090493). They were housed in a temperature‐controlled, pathogen‐free environment on a 12‐h light/dark cycle and had ad libitum access to food and water. The number of animals used for each experiment was depicted in each figure.

### 2.5 Preparation of Hydrogen‐Enriched HA Dressing

Sodium hyaluronate was dissolved in pure water to prepare a 1% HA, which was then applied to medical gauze and secured to the wound site on the rats. The rats were subsequently placed in a 4% hydrogen gas‐riched container for 2 h to allow the HA to absorb sufficient hydrogen. The 4% hydrogen gas was supplied by our self‐made device, where hydrogen from a hydrogen generator was mixed with air from an air generator in a plastic box. A flow meter was used to regulate the flow rates of each gas, and the hydrogen concentration in the mixed gas was measured using a hydrogen detector (XP‐3140, New Cosmos Electric Co. Ltd., Japan). The total flow rate was about 3 L/min.

### 2.6 Establishment and Administration of Diabetic Foot Ulcer (DFU) Model

Rats were randomly divided into four groups: Control group (Con), DFU model group, HA group (DFU + HA), and hydrogen‐enriched HA dressing group (DFU + HA + H_2_). The control group received a standard diet, while the other groups were fed a high‐fat diet for 4 weeks. After a 12‐h fast, the rats were intraperitoneally injected with a single dose (45 mg/kg) of streptozotocin (STZ) to induce diabetes. The control rats were administered an equivalent volume of citrate buffer. Rats exhibiting blood glucose levels ≥ 16.65 mmol/L 1 week posttreatment were classified as diabetic. Blood samples were collected from the caudal vein and analyzed for plasma glucose using a portable blood glucometer (On Call, China).

An 8‐mm skin punch device was used to create wounds on the feet of all groups, followed by respective treatment. Physiological saline‐soaked medical gauze for Con and DFU groups, HA‐soaked medical gauze for DFU + HA group, and hydrogen‐enriched HA dressing for DFU + HA + H_2_ group. Wound healing was monitored on Days 1, 5, 9, and 14, with healing percentage calculated. On Day 14, rats were euthanized and wound samples were collected for protein extraction and histological analysis.

### 2.7 Histological Analysis

Rat wound tissues were immersed in 4% paraformaldehyde for fixation, followed by graded ethanol dehydration, embedded in paraffin, and sectioned serially into 5 μm thick slices. After dewaxing in xylene, the sections were rehydrated through a graded ethanol series. For hematoxylin and eosin (H&E) staining, sections were sequentially stained with H&E, dehydrated, and then sealed with neutral gum. For Masson's trichrome staining (Solarbio, China), the samples were sequentially stained with Ponceau red, phosphomolybdic acid, and aniline blue, followed by dehydration and sealing with neutral resin. The stained sections were observed under an optical microscope (Olympus, Japan).

### 2.8 ELISA Detection of Inflammatory Cytokines

Following the manufacturer's instructions, the levels of TNF‐α, IL‐1β, and IL‐6 in rat serum were measured using ELISA kits (Enzyme‐linked Biotechnology, China) with an Infinite F200 microplate reader (Tecan, Switzerland).

### 2.9 Cells Culture and Treatment

HFF‐1 cells provided by Shanghai cell bank of Chinese Academy of Sciences (Shanghai, China) were cultured in Dulbecco's modified Eagle's medium (DMEM; Gibco, USA) supplemented with 15% fetal bovine serum (FBS; Excell). Cells were maintained in a humidified atmosphere containing 5% CO_2_ at 37°C. To induce cell injury, HFF‐1 cells were placed in DMEM containing HG (75 mM) for 24 h. Sodium hyaluronate powder was dissolved in pure water to a final concentration of 1% and subsequently mixed with high‐glucose culture medium. The cells were placed in a specialized hydrogen incubator (4% H_2_, 50% N_2_, 40% O_2_, and 10% CO_2_) for 24 h.

### 2.10 Immunofluorescence Staining Assay

Paraffin sections underwent antigen retrieval, blocked with 5% normal donkey serum, and incubated with CD31 primary antibody (Proteintech, 11 265‐1‐AP, 1:200) at 4°C overnight. Sections were then incubated with Alexa Fluor 488‐conjugated goat anti‐rabbit antibody (Invitrogen, 2 μg/mL) for 1 h. Slides were mounted with the mounting medium and viewed using a fluorescence microscope (Olympus, Tokyo, Japan).

### 2.11 Cell Viability Assay

HFF‐1 cell viability was assessed using Cell Counting Kit‐8 (CCK‐8; YiShan Biotech, China). Briefly, HFF‐1 cells were seeded in 96‐well plates (6 × 10^3^ cells per well). After cell attachment, the cells were treated with the conditions described above and incubated for 24 h at 37°C in a 5% CO_2_ incubator or in a 4% hydrogen gas incubator. Afterward, 10 μL of CCK‐8 reagent was added to each well, and the cells were incubated for an additional 1 h. The optical density at 450 nm was measured using a BioTek Synergy H1 microplate reader (Molecular Devices, Agilent, USA).

### 2.12 Mitochondrial Membrane Potential (ΔΨ_m_ ) Detection

A mitochondrial membrane potential assay kit with JC‐1 (Beyotime, China) was used to test ΔΨ_m_. According to the manufacturer's instructions, dilute the JC‐1 working solution 200 times for use in staining cells for 20 min, and then wash the cells twice with staining buffer. Next, incubate with Hoechst 33342 for 5 min to stain the nuclei. Finally, capture fluorescent images using a confocal microscope (Nikon, Japan).

### 2.13 Cell Apoptosis Assay

According to the manufacturer's protocol (Multi Sciences, China), HFF‐1 cell apoptosis was quantitatively assessed using the Annexin V‐FITC/propidium iodide (PI) dual staining method. After the aforementioned treatments, cells were centrifuged, washed with PBS, and resuspended in binding buffer. The cells were then incubated with Annexin V‐FITC and PI solution at room temperature in the dark for 5 min. Samples were analyzed using a NovoCyte flow cytometer (ACEA, CA, USA) with Diva software.

### 2.14 Detection of ROS Levels

HFF‐1 cells were inoculated in six‐well plates (1 × 10^6^ per well) and treated under different conditions. DCFH‐DA (Beyotime, China) was used to detect intracellular ROS production. DCFH‐DA was diluted at 1:1000 in serum‐free medium, and 2 mL of DCFH‐DA (10 μM) was added to each well. The cells were incubated at 37°C for 20 min under dark conditions and washed with the serum‐free medium three times to remove unabsorbed DCFH‐DA fully. ROS in cells was detected by a fluorescence microscope (Olympus, Japan). Image J software was used to quantify the fluorescence intensity of the images.

### 2.15 Cell Migration Assay

The migratory capacity of HFF‐1 cells was assessed using a cell scratch assay. Briefly, treated cells were implanted into six‐well plates (1 × 10^6^ per well). After the cells reached approximately 80% confluence, a linear scratch was introduced into the monolayer using a sterile pipette tip. The wells were then washed three times with PBS to remove any floating cells, and medium containing 1% FBS, along with the indicated treatments, was added. Photographs of the scratch were taken at 0, 12, and 24 h to monitor cell migration using an inverted optical microscope (Olympus, Japan). Migration areas were quantified using ImageJ software.

### 2.16 Western Blotting

RIPA lysis buffer (Beyotime, China) was used to isolate proteins from HFF‐1 cells and rat tissues. After quantifying the protein concentration using the BCA method (MeilunBIO, China), 20 μg of protein was loaded onto SDS‐PAGE and transferred to a PVDF membrane (Millipore, USA) via electroblotting. The membrane was incubated in 5% nonfat milk for 2 h, followed by overnight incubation with the primary antibody at 4°C. On the following day, the membrane was incubated with HRP‐conjugated mouse (Proteintech, SA00001‐1, 1:5000) or rabbit (Proteintech, SA00001‐2, 1:5000) secondary antibody at room temperature for 2 h. Finally, the membrane was exposed to an ECL chemiluminescent substrate kit (EpiZyme, China) to visualize protein bands, and band intensity was quantified using ImageJ software. GAPDH was used as the loading control. The primary antibody details are as follows: VEGFA (Proteintech, 19003‐1‐AP, 1:5000), ICAM‐1 (Proteintech, 10020‐1‐AP, 1:2500), VCAM‐1 (Affinity, DF6082, 1:1500), SIRT3 (Proteintech, 10099‐1‐AP, 1:4500), FOXO3A (Proteintech, 10849‐1‐AP, 1:6000), PINK1 (Affinity, DF7742, 1:2000), PARKIN (Affinity, AF0235, 1:1500), P62 (Affinity, DF5384, 1:1500), LC3B (Proteintech, 14600‐1‐AP, 1:2500), SOD2 (Affinity, AF5144, 1:1250), AC‐SOD2 (Zenbio, R30160, 1:750), GAPDH (Proteintech, 60004‐1‐AP, 1:10000).

### 2.17 Statistical Analysis

Image information was quantified using ImageJ software, and the statistical graphs of the experimental data were generated using GraphPad Prism 8 software. For comparisons of two groups, an unpaired two‐tailed Student's *t*‐test was used. For the comparisons of multiple groups, the one‐way ANOVA analysis was performed. All data were provided in the form of mean ± standard deviation (SD). *p* < 0.05 represents statistically significant.

## 3 Results

### 3.1 Hydrogen‐Rich Saline Combined With VSD Promotes DFU Healing in Patients

**FIGURE 1:**
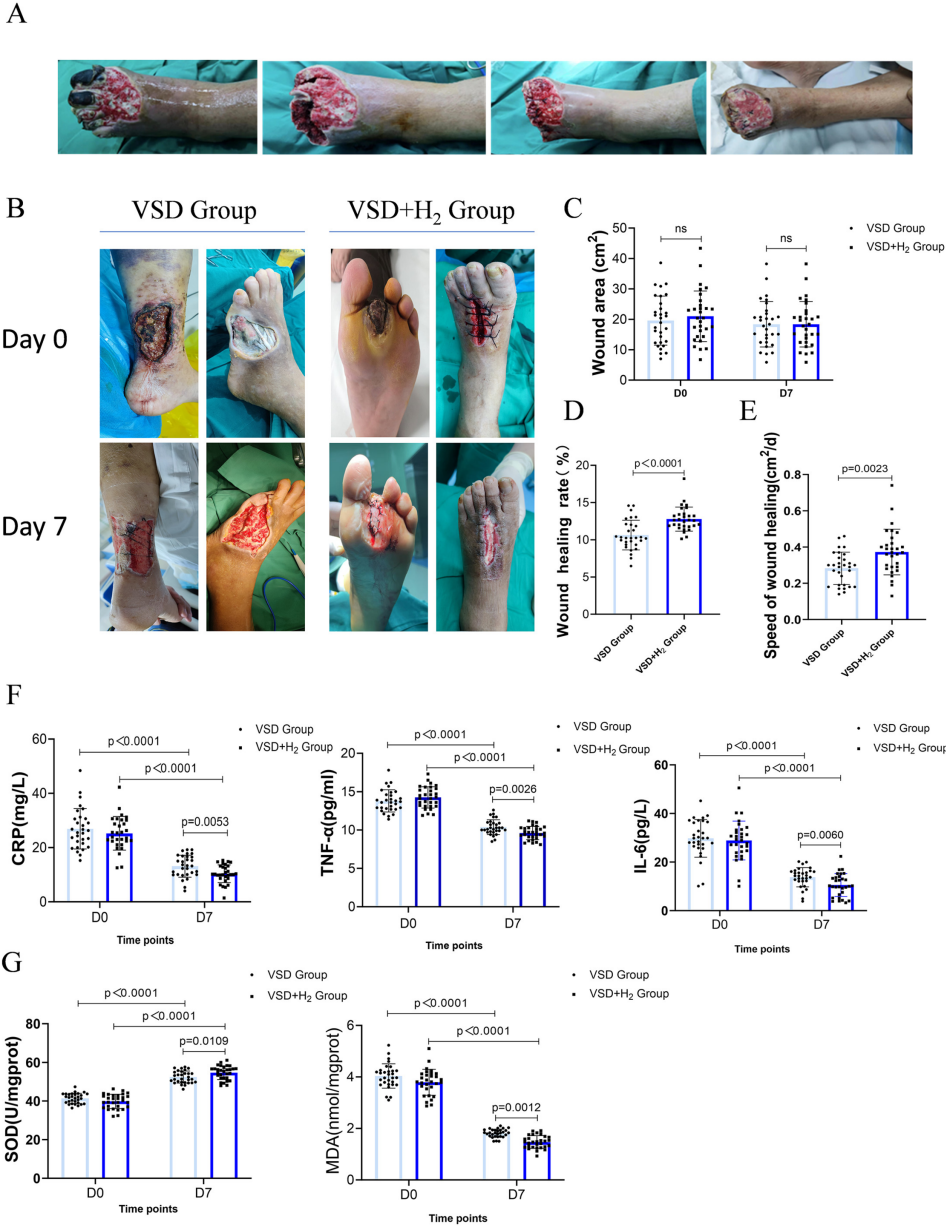
Hydrogen‐rich saline combined with VSD promotes DFU healing in patients (A) Wound appearance at different stages of treatment combining VSD and hydrogen‐rich saline. (B) Real‐life images of wound healing outcomes in two patient groups before and after treatment. (C–E) Wound area, healing rate, and healing speed in patients (*n* = 30). (F) Serum levels of CRP, TNF‐α, and IL‐6 in patients before and after treatment (*n* = 30). (G) Levels of oxidative markers MDA and SOD in wound tissues of patients before and after treatment (*n* = 30). Data are expressed as means ± SD. Results were compared with an unpaired *t* test.

In this study, we firstly performed preoperative disinfection using povidone‐iodine and irrigation with hydrogen peroxide, then necrotic tissues were debrided following suturing of the foot tissue; finally, effects of VSD and hydrogen‐rich saline on wound repair were investigated by monitoring the process of wound healing with related measurements (Figure 1A).

Wound imaging showed there was no significant difference in wound area between the two groups before and after treatment, which may be attributed to the small sample size and short treatment duration (Figure 1B,C). However, the VSD + H_2_ group had a higher wound healing rate and faster healing speed compared to the VSD group (Figure 1D,E). Additionally, multivariate analysis of covariance (ANCOVA) adjusting for baseline differences confirmed that the treatment effect remained highly significant (Table S3). Furthermore, the VSD + H_2_ group demonstrated superior efficacy in reducing serum inflammatory markers compared to the VSD group (Figure 1F), with significantly lower malondialdehyde (MDA) levels and higher superoxide dismutase (SOD) activity at the wound site (Figure 1G). In conclusion, these findings suggest that hydrogen‐rich saline combined with VSD enhances DFU healing through its anti‐inflammatory and antioxidant properties.

Additionally, no obvious abnormalities were observed in routine blood tests, urine tests, liver and kidney function, and other laboratory indicators after treatment. No significant differences between the two groups were found in baseline, including gender, age, DFU duration, HbA1c levels, or Wagner grade (Table S2).

### 3.2 Hydrogen‐Enriched HA Dressing Accelerates DFU Healing

**FIGURE 2:**
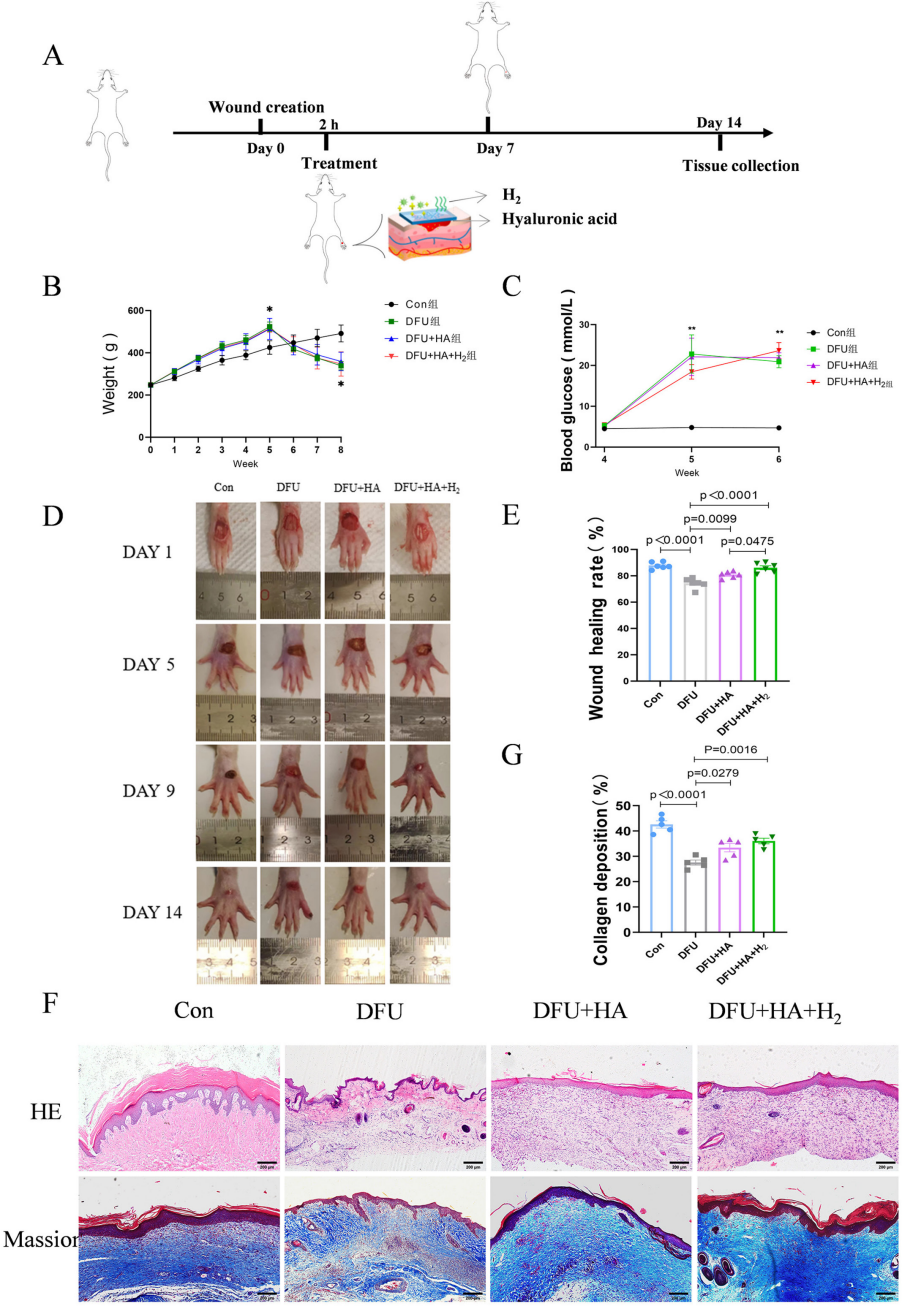
Impact of hydrogen‐enriched hyaluronic acid dressings on wound healing in diabetic foot ulcer rats. (A) Establishment of the diabetic wound model and treatment procedure. (B) Body weight level. (C) Blood glucose level. (D) Digital images of diabetic wounds. (E) Corresponding wound size statistic (*n* = 6). (F) The representative images of HE and Masson staining showing the tissue remodeling 14 days postwounding (scale bar = 200 μm). (G) Quantification of the collagen deposition in different groups (*n* = 5).

The diabetic wound model was established in SD rats by the aforementioned method, followed by daily treatment of the wounds for 2 h with different therapies (Figure 2A). Rats in the diabetic group experienced significant weight loss (Figure 2B), and a sharp increase in blood glucose levels followed by stabilization (Figure 2C). From the representative images of wounds during treatment in Figure 2D,E, the wound closure rate in the DFU + HA + H_2_ group was significantly higher than that in the other groups, approaching that of the normal control group without diabetes. In order to delineate the individual contributions and assess potential synergy, additional animal groups receiving hydrogen or HA monotherapy were included (Figure S1A,B). Consistent with a synergistic mechanism, the combined hydrogen‐rich HA treatment yielded significantly greater wound closure rates than either component alone.

To further investigate the impact of the hydrogen‐enriched HA dressing on wound healing, we conducted histological analysis using H&E and Masson‐stained sections. The wounds in the DFU group displayed epidermis damage and inflammatory cell invasion. The DFU + HA group exhibited relatively immature granulation tissue and an incomplete epidermis. Meanwhile, the wound managed with hydrogen‐enriched HA dressing experienced better recovery, featuring densely arranged granulation tissue and relatively thicker epidermis (Figure 2F). Masson staining revealed an increase in collagen fibers in the HG + HA + H_2_ group, promoting the deposition of collagen and the regeneration of the ECM (Figure 2G). Therefore, these results suggest that the hydrogen‐enriched HA dressing accelerates diabetic wound healing.

### 3.3 Hydrogen‐Enriched HA Dressing Reduces Inflammatory Factors and Promotes Angiogenesis in Vivo in Animal

**FIGURE 3:**
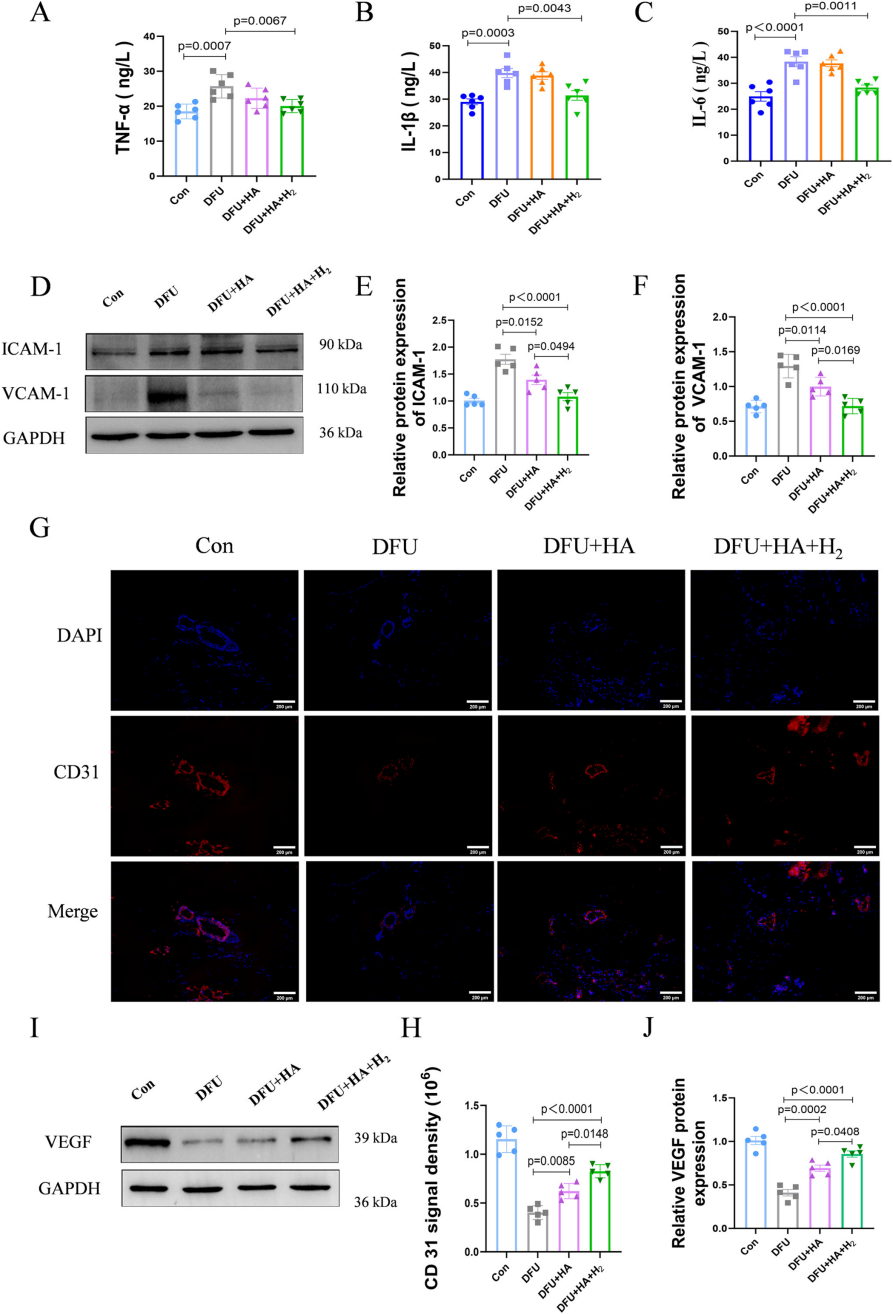
Hydrogen‐enriched hyaluronic acid dressing reduces inflammatory factors and promotes angiogenesis in animal vivo. (A–C) ELISA detection of TNF‐α, IL‐1β, and IL‐6 levels in rat serum (*n* = 6). (D–F) Western Blot was used to detect ICAM‐1 and VCAM‐1; the bands represent protein levels (*n* = 5). (G) Immunofluorescence staining images of CD31 (scale bar = 200 μm). (H) Statistics of the immunofluorescence experiment. The data represent mean ± SD (*n* = 5). (I, J) Western Blot was used to detect VEGF (*n* = 5).

In histological analysis, we observed a significant reduction in inflammatory factors and neovascularization at the wound site following treatment with the hydrogen‐enriched HA dressing. Consequently, we further investigate the effect of the hydrogen‐enriched HA dressing on the diabetic wound by ELISA and western blot. We found that the serum levels of TNF‐α, IL‐1β, and IL‐6 were significantly reduced after treatment with the hydrogen‐enriched HA dressing (Figure 3A–C). Additionally, protein analysis of skin tissue indicated that the expression of related inflammatory factors was also significantly decreased following treatment (Figure 3D–F).

To evaluate wound angiogenesis, CD31 was employed to characterize wound blood vessels. The number of new blood vessels in each group was systematically evaluated via immunofluorescence. Results showed that the number of blood vessels in the DFU + HA + H_2_ group was significantly higher than in both the DFU group and the DFU + HA group (Figure 3G,H). Furthermore, the protein expression of vascular endothelial growth factor (VEGF) was also significantly elevated (Figure 3I,J). Overall, these findings suggest that the hydrogen‐enriched HA dressing inhibits inflammation and enhances angiogenesis during wound healing.

### 3.4 Hydrogen‐Enriched HA Dressing Promotes the Expression of SIRT3 and Affects Mitophagy in Vivo in Animal

**FIGURE 4:**
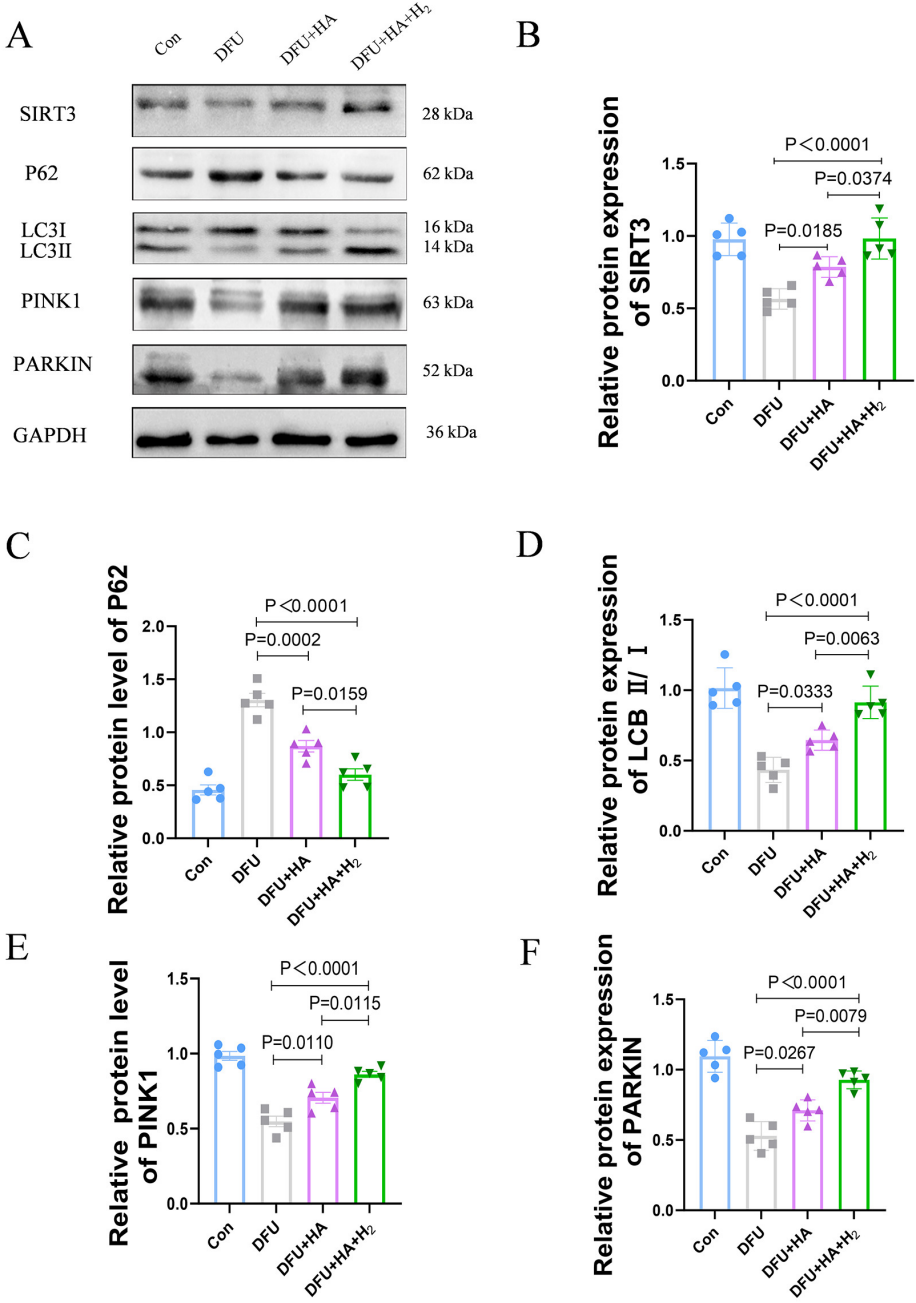
Hydrogen‐enriched hyaluronic acid dressing promotes the expression of SIRT3 and affects mitochondrial autophagy in animal vivo. (A–F) Western blot analysis of P62, LC3II/I, PINK1, PARKIN, and SIRT3 expression in the skin around the wound on the 14th day after treatment (*n* = 5).

To further explore the mechanism underlying the hydrogen‐enriched HA dressing‐increased wound repair, we investigate whether SIRT3 and mitophagy are involved in this process based on previous publications. Through western blot, we confirmed that the expression of SIRT3 in the DFU group was decreased, while the treatment with the hydrogen‐enriched HA dressing restored the protein levels of SIRT3. Mitophagy refers to the selective degradation of mitochondria via autophagy, serving as a protective mechanism for cells under environmental stress. Western blot results showed that, compared to the DFU group, the levels of autophagy and mitochondrial autophagy markers were elevated in the DFU + HA + H_2_ group, indicating that the treatment promoted mitophagy (Figure 4A–F).

### 3.5 HA and Hydrogen Synergistically Reduce Damage to Human Fibroblasts Induced by High Glucose and Promote the Expression of SIRT3

Dermal fibroblasts are the main repair cells involved in wound healing. In DFU wounds, prolonged local hyperglycemia, hypoxia, and chronic inflammation severely impair processes such as fibroblast proliferation, migration, and differentiation, making chronic wounds difficult to heal. Therefore, we selected these cells for in vitro experiments to investigate the underlying mechanisms. We first tested the impacts of different concentrations of glucose on cell viability using the CCK‐8 assay, ultimately choosing 75 mM as high glucose levels (Figure S2A). Importantly, this effect was not attributable to osmotic stress, as confirmed by an isosmotic mannitol control which showed no significant impact on cell viability (Figure S2B). To investigate the combined effects of HA and hydrogen treatment, we observed the cell morphology and evaluated cell viability. We found that high glucose levels induced cell damage while the synergistic effect of HA and hydrogen significantly protected the growth of cells (Figure S3A,B). Moreover, high glucose levels also decreased the expression of SIRT3 within the cells, which is restored by the treatments (Figure S4A,B). These findings exhibit that HA and hydrogen synergistically inhibit high glucose‐induced cell death with increased expression of SIRT3 in human fibroblasts.

### 3.6 HA and Hydrogen Synergistically Enhance High Glucose‐Impaired Mitophagy and Reduce Cell Apoptosis via SIRT3

**FIGURE 5:**
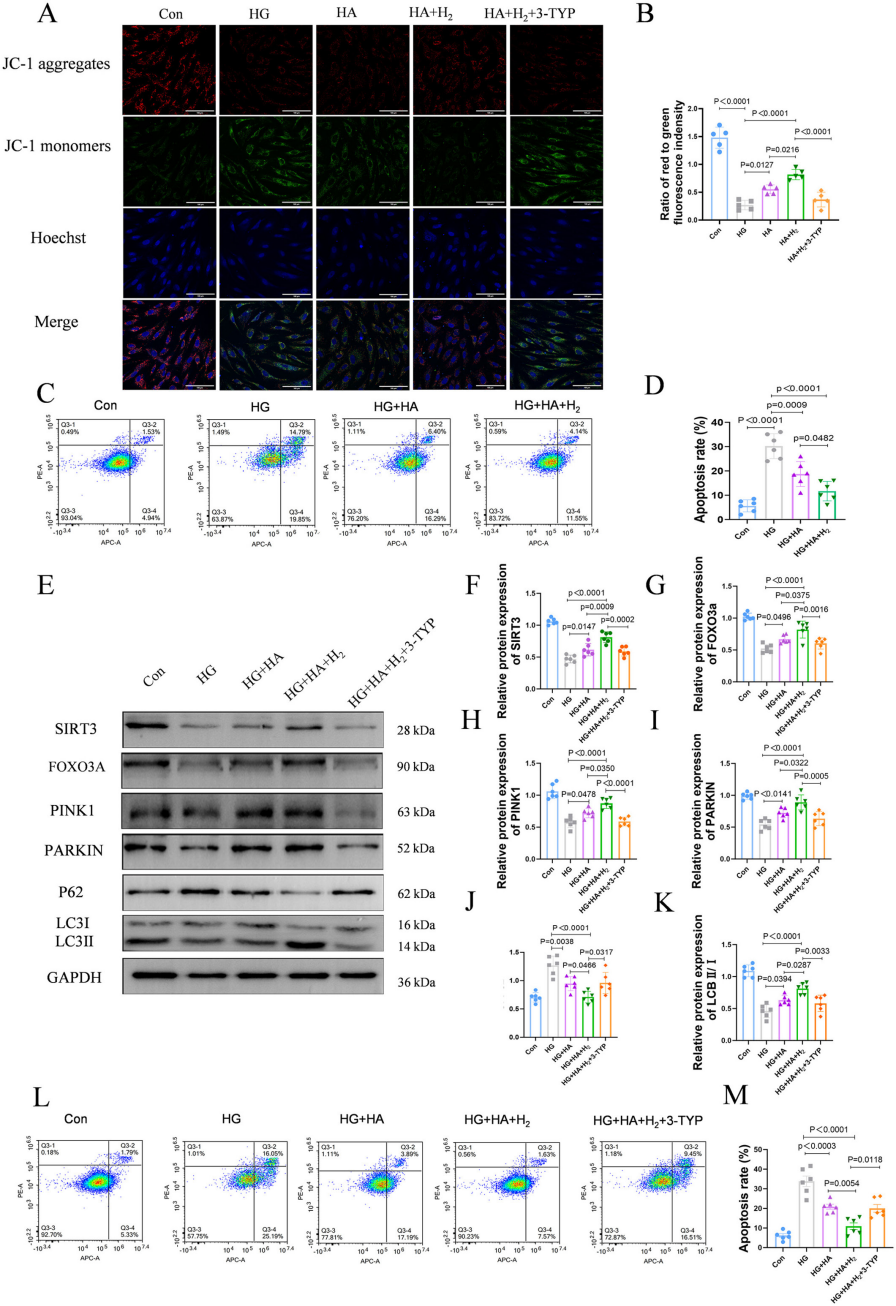
Hyaluronic acid and hydrogen synergistically protect mitochondrial function and reduce cell apoptosis through SIRT3. (A) JC‐1 signal in HFF‐1 cells was examined by fluorescence confocal microscopy. Cells were labeled with Hoechst to show the nucleus (blue) and stained with JC‐1 to show the mitochondria. Double staining of cells with JC‐1 is shown: green for monomers, red for aggregates (scale bar = 100 μm, *n* = 5). (B) Mean optical density of the ratio of aggregate to monomer (*n* = 5). (C, D) Flow cytometry for the detection of cell apoptosis by Annexin V/PI staining. The data are presented as the means ± SD (*n* = 6). (E–K) Western Blot was used to detect *SIRT3*, FOXO3A, PINK1, PARKIN, P62 and LC3 II/I. The bands represent protein levels (*n* = 6). (L, M) Flow cytometry for the detection of cell apoptosis by Annexin V/PI staining. The data are presented as the means ± SD (*n* = 6).

Previous reports suggest that SIRT3 overexpression may affect apoptosis by regulating mitophagy via the FOXO3A/PINK1‐PARKIN signaling pathway [20]. Simultaneously, we detected via flow cytometry that high glucose induces apoptosis in skin fibroblasts, while treatment attenuated apoptosis (Figure 5C,D).

Subsequently, we investigated whether high glucose‐induced apoptosis was regulated by the FOXO3A/PINK1‐PARKIN signaling pathway. Impaired mitophagy often accompanies mitochondrial damage. Therefore, we firstly detected changes in the mitochondrial membrane potential as an indicator of mitochondrial damage. The protective effect of hydrogen and HA on mitochondria in HFF‐1 cells stimulated by high glucose was assessed by JC‐1 staining. It was found that cells treated with high glucose mainly contain JC‐1 in their monomeric form, while after treatment with hydrogen and HA, JC‐1 primarily exists in its aggregated form on mitochondria (Figure 5A,B). Meanwhile, the combined application demonstrated superior efficacy in restoring mitochondrial membrane potential compared to monotherapy with either HA or hydrogen alone (Figure S5A,B). Interestingly, high glucose‐induced mitochondrial damage was reversed by 3‐TYP, a SIRT3‐specific inhibitor preventing proper deacetylation of SIRT3 substrates. Thus, we speculate that the synergistic effects of hydrogen and HA regulate mitochondrial autophagy via SIRT3 and its downstream pathways, thereby affecting cell apoptosis. Western blot was conducted to detect relevant proteins in this pathway. Compared to the HG group, the HG + HA + H_2_ group showed a significant increase in the expression levels of SIRT3, FOXO3A, PINK1, PARKIN, P62, and the LC3II/LC3I ratio, indicating the emergence of mitophagy. Following the addition of 3‐TYP, the level of mitochondrial autophagy decreased (Figure 5E–K). To establish a direct causal link, SIRT3 expression was specifically knocked down using siRNA in the treated group. This genetic intervention markedly attenuated the upregulation of downstream FOXO3A and PINK1‐Parkin pathway proteins, confirming that SIRT3 activation is essential for the observed pro‐mitophagic effect (Figure S6A–G). Apoptosis detection showed that the treatment effect was also inhibited following the addition of the inhibitor (Figure 5L,M). These results revealed that inhibition of SIRT3 could block SIRT3‐mediated activation of the FOXO3A/PINK1‐PARKIN pathway, thereby restraining mitophagy. This finding further confirmed that hydrogen and HA synergistically regulate mitophagy and inhibit apoptosis by activating the FOXO3A/PINK1‐PARKIN pathway through SIRT3.

### 3.7 HA and Hydrogen Synergistically Reduce Oxidative Stress and Promote Cell Migration via the SIRT3/SOD2 Pathway

**FIGURE 6:**
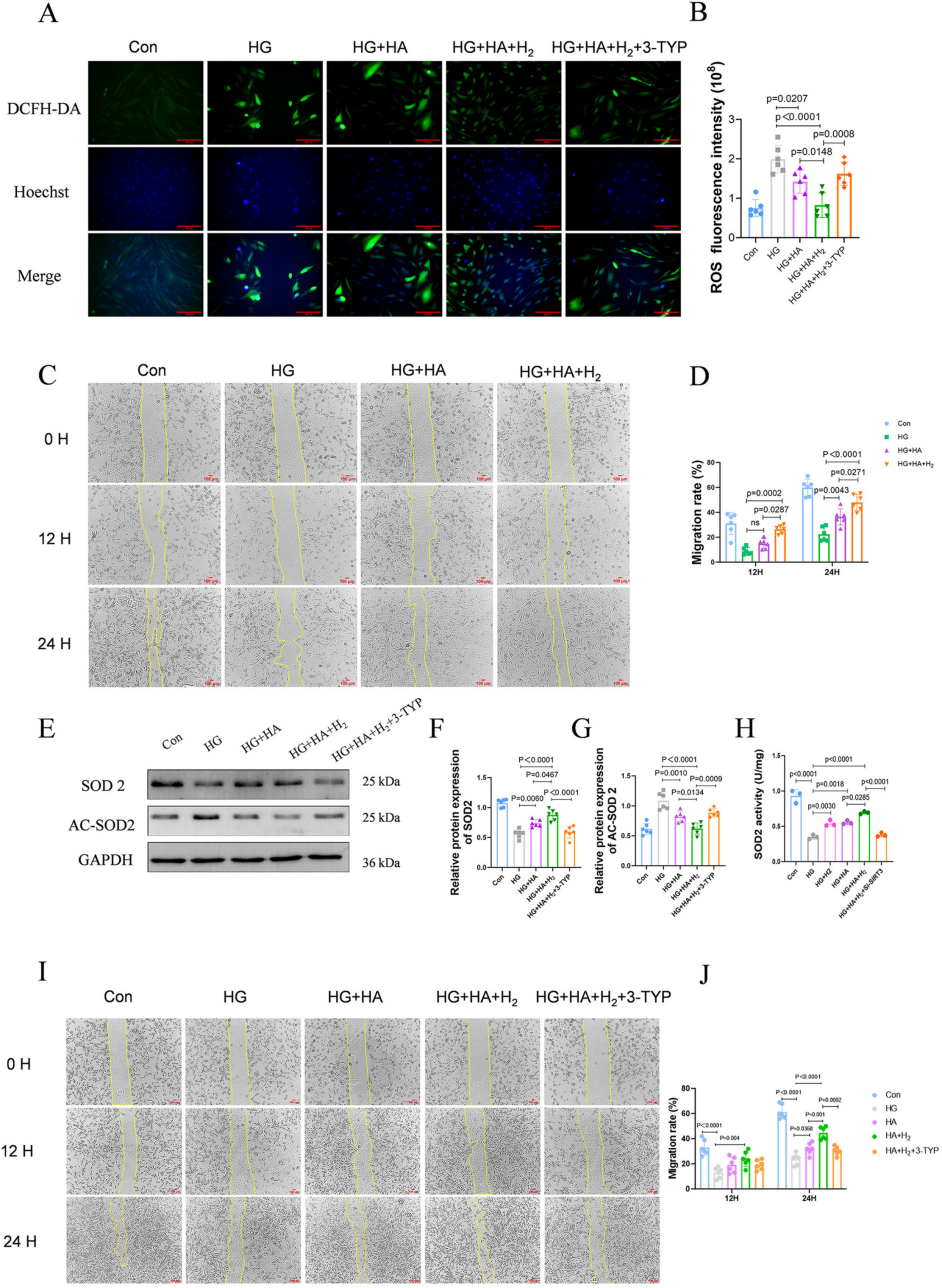
The synergistic effect of hyaluronic acid and hydrogen reduces oxidative stress and promotes cell migration via the SIRT3/SOD2 pathway. (A, B) The generation of intracellular ROS production was tested by DCFH‐DA probe‐based immunofluorescence staining (scale bar = 100 μm, *n* = 6). (C, D) The effect of the synergistic effects of hyaluronic acid and hydrogen on the migration of HFF‐1 cells in a high‐glucose environment was detected by scratch wound assays (scale bar = 100 μm, *n* = 6). (E–G) Western Blot was used to detect SIRT3, SOD2, and AC‐SOD2; the bands represent protein levels (*n* = 6). (H) Measurement of SOD2 enzymatic activity (*n* = 3). (I, J) The effect of adding 3‐TYP to the treatment group on the migration of HFF‐1 cells in a high‐glucose environment was detected by scratch wound assay (scale bar = 100 μm, *n* = 6).

Under high glucose conditions, excessive ROS are generated, resulting in oxidative damage. HA enhances cellular hydration and creates a favorable microenvironment, which decreases oxidative stress. Hydrogen is a potent antioxidant, further mitigating ROS production. To monitor intracellular ROS generation, we employed the DCFH‐DA fluorescence probe‐based immunostaining method. The results demonstrated that the synergistic effect of HA and hydrogen significantly reduced intracellular ROS in fibroblasts exposed to high glucose (Figure 6A,B).

In wound healing, oxidative stress impairs cell migration, delaying healing and promoting chronic wound formation [25]. Thus, we employed the scratch test to investigate the impact of the synergistic effects of HA and H_2_ on the migratory ability of fibroblasts. Results from the scratch test demonstrated that the migratory ability of fibroblasts was significantly increased with the HG + HA + H_2_ group at 12 and 24 h compared to the HG group (Figure 6C,D). Meanwhile, endothelial cells treated with HA‐Hydrogen exhibited a pronounced pro‐angiogenic response in the tube formation assay, confirming the functional role of the dressing in promoting vascular structure assembly (Figure S7A–E).

Activation of SIRT3 has been shown to upregulate SOD2, thereby facilitating the removal of superoxide and reducing oxidative injury [26]. Therefore, we speculate that the synergistic effect of HA and hydrogen reduces oxidative stress and promotes cell migration via the SIRT3/SOD2 pathway. To validate this hypothesis, the exogenous ROS donor H_2_O_2_ was added to the treatment group. We observed that it reversed both the ROS‐scavenging effect and the pro‐migratory therapeutic outcome (Figure S8A–D), clearly demonstrating that the attenuation of oxidative stress is a key factor in improving cell migration. Meanwhile, western blot analysis confirmed that the synergistic effects of hydrogen and HA promote the deacetylation of SOD2 by SIRT3 (Figure 6E–G). Critically, the rise in SOD2 protein was concomitant with a marked increase in its enzymatic activity, demonstrating a specific dependence on SIRT3‐mediated deacetylation and hallmarks its genuine functional activation (Figure 6H). Furthermore, we employed the SIRT3 inhibitor 3‐TYP and found that HA and hydrogen decreased ROS clearance capacity (Figure 6A,B), inhibited the deacetylation of SOD2 by SIRT3, and diminished cell migration (Figure 6I,J). Collectively, these results demonstrate that the synergistic therapy of HA and hydrogen mitigates high glucose‐induced oxidative stress via the SIRT3/SOD2 pathway, thereby promoting cell migration.

## 4 Discussion

As the global population ages, chronic wounds, particularly diabetic ulcers, are expected to remain a significant challenge in terms of medical, social, and economic impact [27]. Here, the hydrogen‐enriched and HA dressing regulates mitophagy and oxidative stress through SIRT3 and its downstream pathways, including FOXO3A/PINK1‐PARKIN and SOD2, thereby enhancing the repair capacity of skin fibroblasts in order to accelerate the healing of DFU.

In the present study, for clinical applications, VSD technology was employed to achieve comprehensive wound closure, thereby allowing the infused hydrogen‐rich saline to fully exploit its anti‐inflammatory and antioxidant properties. Our findings demonstrated that hydrogen improved wound healing, which in turn indirectly led to a reduction in systemic inflammation. Additionally, medical foam dressings provided an optimal moist environment for wound healing during the clinical trial. In vivo animal experiments, we developed a hydrogen‐enriched HA dressing. HA, a biocompatible natural polymer, has been extensively utilized in wound healing applications. Recent studies have demonstrated that HA hydrogel can accelerate skin wound healing in rats [28]. However, HA alone is limited in modulating the oxidative and inflammatory microenvironment characteristic of diabetic ulcers, particularly, in its inability to restore mitochondrial homeostasis and promote mitophagy. Thus, this dressing not only preserves a moist wound environment, preventing secondary infections, but also effectively encapsulates hydrogen, ensuring its sustained therapeutic effects. Our research findings indicate that the treatment effectively reduced inflammation and enhanced the angiogenic capacity; it is directly supported by the observed reduction in key clinical inflammatory markers, including serum CRP and TNF‐α levels, in patients treated with the dressing compared to the control group. This clinical finding is corroborated by our animal experiments, which showed a concurrent downregulation of pro‐inflammatory cytokines in wound tissues. The pro‐angiogenic effect of the HA‐Hydrogen dressing is another key finding of our study. In vivo experiments demonstrated that treatment with the dressing significantly enhanced the expression of CD31 and VEGF. Moreover, the pro‐angiogenic effect was further validated by the endothelial tube formation assay. Collectively, these data indicate that the HA‐Hydrogen system actively modulates the hyper‐inflammatory state characteristic of diabetic wounds, transitioning the microenvironment from a chronic inflammatory phase toward a regenerative one, ultimately accelerating skin wound healing. Except that, it is found that the expression of SIRT3 was reduced, and mitochondrial autophagy was suppressed in DFU rats. However, treatment with the hydrogen‐enriched HA dressing had resulted in positive modulation. As reported, mitochondria play a critical role in maintaining skin homeostasis. Upregulated mitophagy has been shown to promote tissue restoration. In contrast, downregulated mitophagy leads to tissue inflammation and damage [29]. SIRT3, as the primary mitochondrial deacetylase, plays a central role in maintaining mitochondrial function and cellular health by regulating various signaling pathways [18]. These are shown by our animal study.

Some studies have reported that hyperglycemia directly affects the activity of keratinocytes and fibroblasts, inducing changes in protein synthesis, apoptosis, and migration, along with increased oxidative stress. These alterations can lead to insufficient angiogenesis and the onset of chronic inflammation, ultimately damaging the skin's barrier function [30, 31, 32, 33, 34]. In vitro experiments, significant morphological damage and cell apoptosis were observed under high glucose conditions. However, the negative effects were effectively rescued by synergistic treatment of HA and hydrogen. It has been proved that SIRT3 can enhance mitochondrial activity and prevent cell apoptosis [35]. Additionally, Urolithin A has been found to reduce fibroblast apoptosis by modulating mitochondrial autophagy via SIRT3, thus protecting cells from photoaging [36]. Based on these discoveries, we assume that the hydrogen‐enriched HA dressing regulates mitochondrial autophagy through SIRT3 and its downstream pathways, leading to reduced apoptosis. In line with this hypothesis, our results indicate that SIRT3 expression is reduced in cells treated with high glucose, but cotreatment with hydrogen and HA significantly elevated SIRT3 levels and led to the activation of FOXO3A. FOXO3A, a transcription factor involved in multiple cellular processes, has been shown to protect cells from apoptosis induced by oxidative stress [37]. FOXO3A is also a key regulator of mitophagy, and SIRT3 can activate FOXO3A expression, suggesting that SIRT3 and FOXO3A form a potential mitochondrial signaling cascade [38]. Previous studies have further demonstrated that PINK1 is an important downstream mediator of FOXO3A, and the activation of FOXO3A can upregulate PINK1, thereby modulating cell apoptosis [39]. When we applied the specific SIRT3 inhibitor 3‐TYP, the activation of FOXO3A was inhibited, which in turn suppressed mitophagy, resulting in increased fibroblast apoptosis. Furthermore, we employed siRNA to knock down SIRT3 expression and found that the protein levels of downstream pathway genes were also reduced. Therefore, the hydrogen‐enriched and HA dressing enhances mitophagy by activating SIRT3 and its downstream FOXO3A/PINK1‐PARKIN pathway, which reduces fibroblast apoptosis in DFU and promotes ulcer healing.

ROS are natural byproducts of cellular respiration. SOD is an important enzyme in the defense against oxidative stress and plays a critical role in maintaining cellular redox balance [40]. Hyperacetylation of SOD2 leads to its inactivation and an increase in oxidative stress [41]. Deacetylation of SOD2 at the Lys68 site is a necessary step for SOD2 activation, which helps reduce ROS accumulation and prevent oxidative stress [42]. Previous studies have shown that under high glucose conditions, the proliferation and migration of endothelial cells are inhibited, impairing angiogenesis. However, exosomes derived from adipose mesenchymal stem cells and stem cells can regulate the expression of SIRT3 and its downstream target SOD2, improving oxidative stress and promoting cell migration to further enhance DFU healing [43]. In our study, the combined treatment of HA and hydrogen significantly increased SIRT3 protein levels in HFF‐1 cells exposed to high glucose. When the specific SIRT3 inhibitor or siRNA was added, it reduced the deacetylation of SOD2, and the positive regulation of oxidative stress and cell migration induced by the combined treatment of HA and hydrogen was reversed. Therefore, these results suggest that the synergistic effect of HA and hydrogen directly stimulates the deacetylase activity of SIRT3, thereby lowering the acetylation level of SOD2 to restore its antioxidant activity. This ultimately prevents cell damage caused by ROS production induced by high glucose, enhancing the migration of HFF‐1 cells.

However, it should also be noted that the current clinical evaluation has limitations. First, the relatively small clinical sample size, along with considerable variation in wound location and initial area that was challenging to standardize, may limit the generalizability of the findings. Furthermore, the treatment and follow‐up period was limited, primarily capturing short‐term efficacy. Finally, while the time window of the “synergistic effect” has not been precisely defined due to the integrated nature of the dressing system, the established continuous exposure protocol and sequential outcome assessments confirm a sustained synergistic interaction. Prior studies indicate that HA aqueous solution can be stored at room temperature for 8 days while maintaining high stability [44]. Moreover, when rats were exposed to hydrogen under airtight conditions, the hydrogen concentration in their blood and tissues peaked at 30 min after inhalation and then gradually declined from 30 to 60 min, and the conclusions remain supported [45]. While these individual properties are established, our study is the first to combine them into a single therapeutic platform. Therefore, future studies with larger sample sizes and longer‐term follow‐up are still needed to further validate the reliability of these findings and assess the durability of the therapeutic effect.

In summary, our study demonstrates that the hydrogen‐enriched HA dressing regulates mitophagy and oxidative stress through SIRT3 and its downstream pathways, including FOXO3A/PINK1‐PARKIN and SOD2, thereby enhancing the repair capacity of skin fibroblasts. Moreover, the new dressing reduces the expression of inflammatory factors, promotes angiogenesis, and increases collagen deposition and remodeling, thereby accelerating skin wound healing. To further contextualize our findings, it is pertinent to discuss emerging precision strategies in wound healing, such as peptide‐based and miRNA‐based therapeutics. These approaches represent a paradigm of target‐specific intervention to improve the wound healing [46, 47, 48, 49, 50]. In contrast, the dressing presented in this study operates through a micro environment‐modulating strategy. While peptide/miRNA therapies aim for high specificity, the hydrogen‐enriched HA dressing offers a holistic modulation of the pathological wound micro environment. These strategies are not mutually exclusive but could be complementary. Thus, the HA‐hydrogen combination offers an innovative and promising therapeutic strategy for the treatment of DFU with considerable clinical potential.

## Author Contributions

Guohua Song, Qingbin Ni, and Xiaolin Ding conceived and supervised the study. Ziyu Xu, Xinyu Cui, and Houbin Chu performed experiments and data analysis. Hao Wan managed by clinical data. Yunbo Xie performed data interpretation and part of the data analysis. Ziyu Xu and Ying Wang written first draft version of the manuscript. All authors discussed and commented on the final article.

## Funding

Funding for this research was provided by the National Natural Science Foundation of China (No. 82370446 and 81873517).

## Ethics Statement

The study was carried out in compliance with the Helsinki Declaration and was authorized by the Second Affiliated Hospital of Shandong First Medical University (No. 2023‐050). Informed consent was obtained from all donors or their guardians. The animal study was approved by the Institutional Animal Research Committee of Shandong First Medical University (No. W202504090493).

## Consent

All participants provided written informed consent, which included explicit authorization for the use of their anonymized data in this study.

## Conflicts of Interest

The authors declare no conflicts of interest.

## Supporting information


**Figure S1:** (A) Digital images of diabetic wounds. (B) Corresponding wound size statistic (*n* = 6).
**Figure S2:** (A) CCK8 assay to assess cell viability in cultures with different glucose DMEM (*n* = 4). (B) CCK‐8 assay to evaluate the effect of 75 mM high‐glucose‐induced osmotic pressure on cell viability (*n* = 6).
**Figure S3:** (A) Cellular morphology observed using an optical microscope. (B) CCK8 assay to evaluate cell viability under different culture conditions (*n* = 5).
**Figure S4:** (A) Western blot results showing the protein levels of SIRT3 in HFF‐1 cells treated with hyaluronic acid and hydrogen in the high‐glucose environment (*n* = 6). (B) Quantified data showing the protein expression of (A).
**Figure S5:** (A) JC‐1 signal in HFF‐1 cells was examined by fluorescence confocal microscopy. Cells were labeled with Hoechst to show the nucleus (blue) and stained with JC‐1 to show the mitochondria. Double staining of cells with JC‐1 is shown: green for monomers, red for aggregates (scale bar = 50 μm, *n* = 3). (B) Mean optical density of the ratio of aggregate to monomer (*n* = 3).
**Figure S6:** (A–G) Western Blot was used to detect *SIRT3*, FOXO3A, PINK1, PARKIN, P62 and LC3 II/I. The bands represent protein levels (*n* = 3).
**Figure S7:** (A) Representative images of tube formation by HUVECs cultured on Matrigel for 6 h under different treatments. (B) number of branch points. (C) number of meshes. (D) number of nodes and (E) total tube length per field (scale bar = 100 μm, *n* = 3).
**Figure S8:** (A, B) The generation of intracellular ROS production was tested by DCFH‐DA probe‐based immunofluorescence staining (scale bar = 50 μm, *n* = 3). (C, D) The effect of the synergistic effects of hyaluronic acid and hydrogen on the migration of HFF‐1 cells in a high‐glucose environment was detected by scratch wound assays (scale bar = 100 μm, *n* = 3).

**Table S1:** Inclusion criteria for clinical samples.
**Table S2:** Comparison of general data between the two groups.
**Table S3:** Analysis of covariance for treatment effect on wound healing rate after adjusting for baseline characteristics.

## Data Availability

The data that supports the findings of this study are available in the Supporting Information of this article.
