# Supplementary material for: Hydrogen‐Enriched Hyaluronic Acid Dressing Ameliorates Diabetic Foot Ulcer via Promoting Mitophagy
Source: J Diabetes. 2026 Mar 29;18(4):e70209. doi: 10.1111/1753-0407.70209 (PMC13112070; doi:10.1111/1753-0407.70209)

**Supplemental Figures**


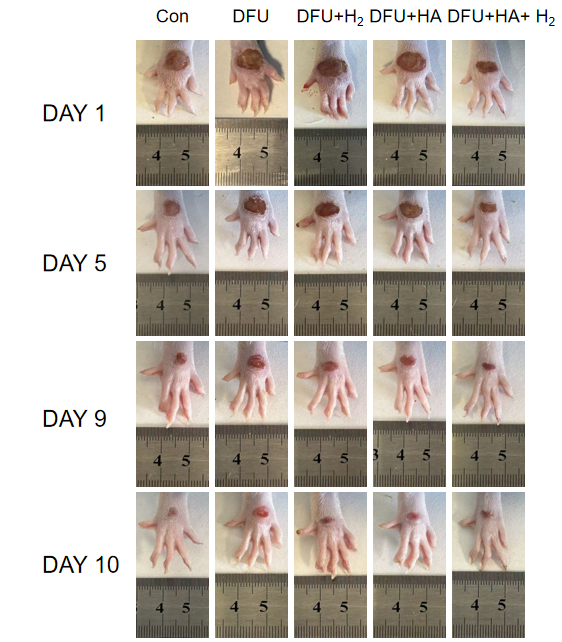


A


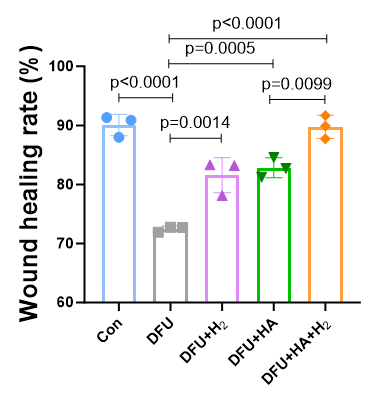


B

**Supplementary Figure 1. (A)** Digital images of diabetic wounds. **(B)** Corresponding wound size statistic (n=6).


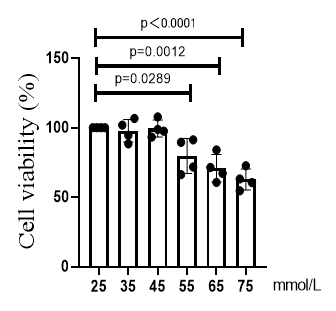


A

B


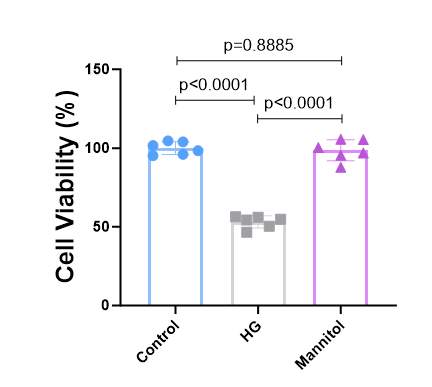


**Supplementary Figure 2. (A)** CCK8 assay to assess cell viability in cultures with different glucose DMEM (n=4). **(B)** CCK-8 assay to evaluate the effect of 75 mM high-glucose-induced osmotic pressure on cell viability (n=6).

B


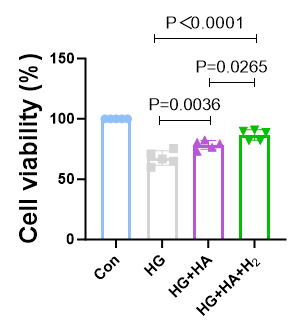


A


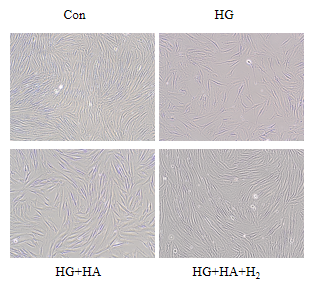


**Supplementary Figure 3. (A)** Cellular morphology observed using an optical microscope. **(B)** CCK8 assay to evaluate cell viability under different culture conditions (n=5).


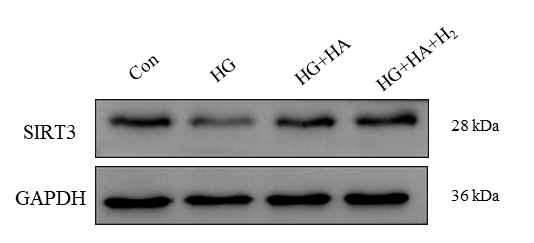


A

B

**Supplementary Figure 4. (A)** Western blot results showing the protein levels of SIRT3 in HFF-1 cells treated with hyaluronic acid and hydrogen in the high-glucose environment (n=6). **(B)** Quantified data showing the protein expression of (A).

A


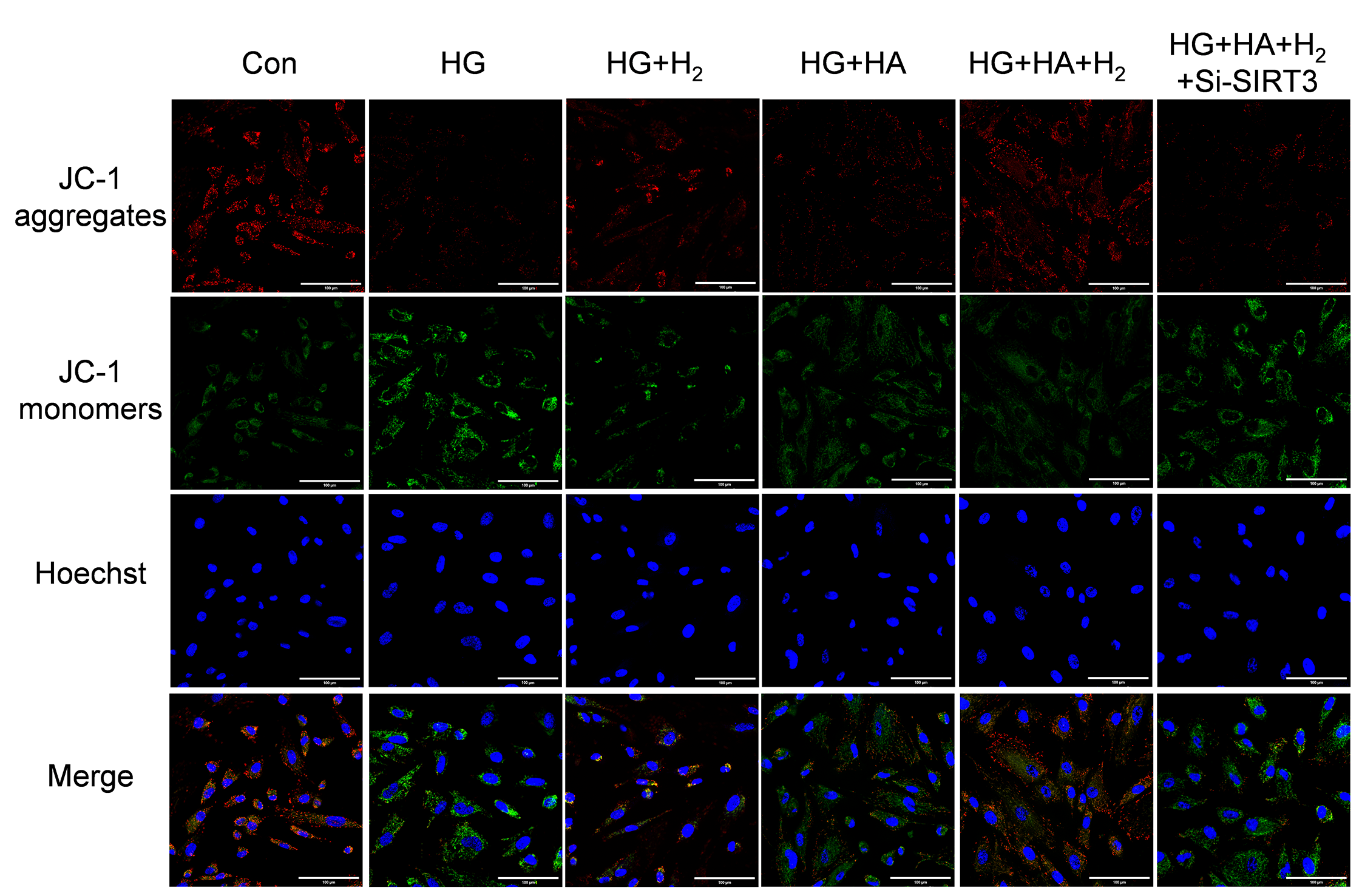


B


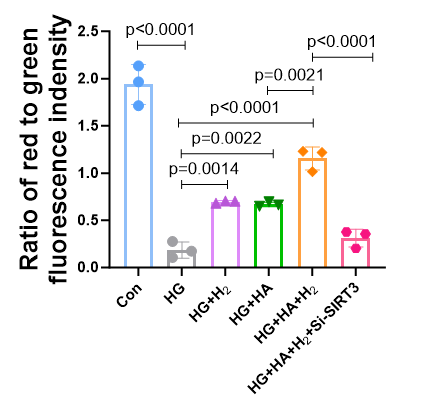


**Supplementary Figure 5. (A)** JC-1 signal in HFF-1 cells was examined by fluorescence confocal microscopy. Cells were labeled with Hoechst to show the nucleus (blue) and stained with JC-1 to show the mitochondria. Double staining of cells with JC-1 is shown: green for monomers, red for aggregates (scale bar = 50 µm, n=3). **(B)** Mean optical density of the ratio of aggregate to monomer (n=3).


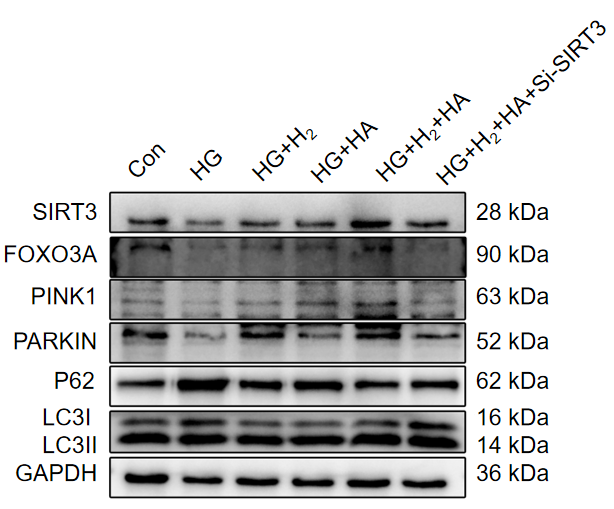


A

B

C

D

E

F

G

**Supplementary Figure 6.** (A-G) Western Blot was used to detect *SIRT3*, FOXO3A, PINK1, PARKIN, P62 and LC3 Ⅱ/Ⅰ. The bands represent protein levels (n=3).


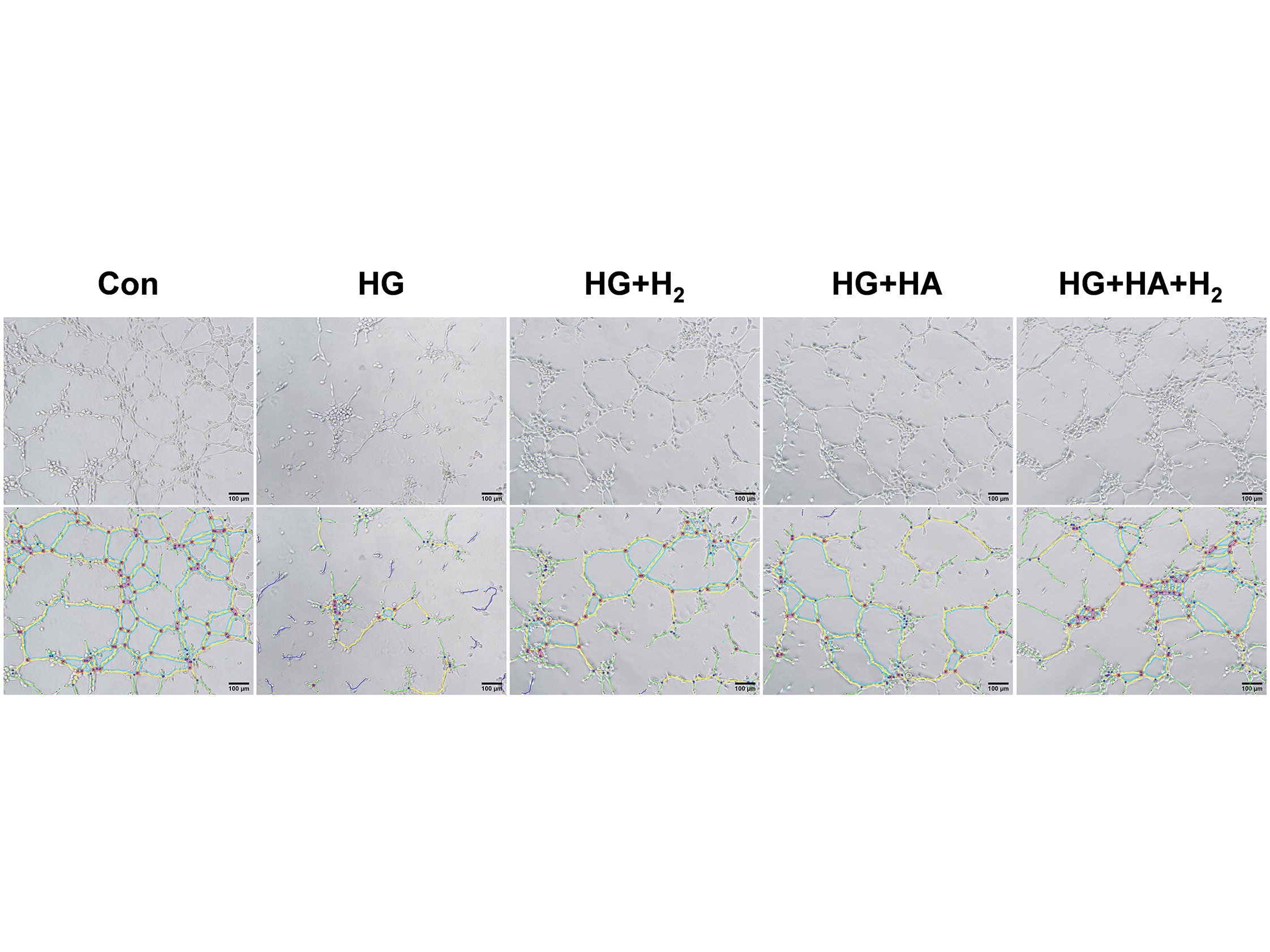


A

B

CC

DC

EC

**Supplementary Figure 7.** **(A)** Representative images of tube formation by HUVECs cultured on Matrigel for 6 hours under different treatments. **(B)** number of branch points. **(C)** number of meshes. **(D)** number of nodes and **(E)** total tube length per field. (scale bar = 100 µm, n=3)

**Supplementary Figure 8. (A-B)** The generation of intracellular ROS production was tested by DCFH-DA probe-based immunofluorescence staining (scale bar = 50 µm, n=3). **(C-D)** The effect of the synergistic effects of hyaluronic acid and hydrogen on the migration of HFF-1 cells in a high-glucose environment was detected by scratch wound assays (scale bar = 100 µm, n=3).


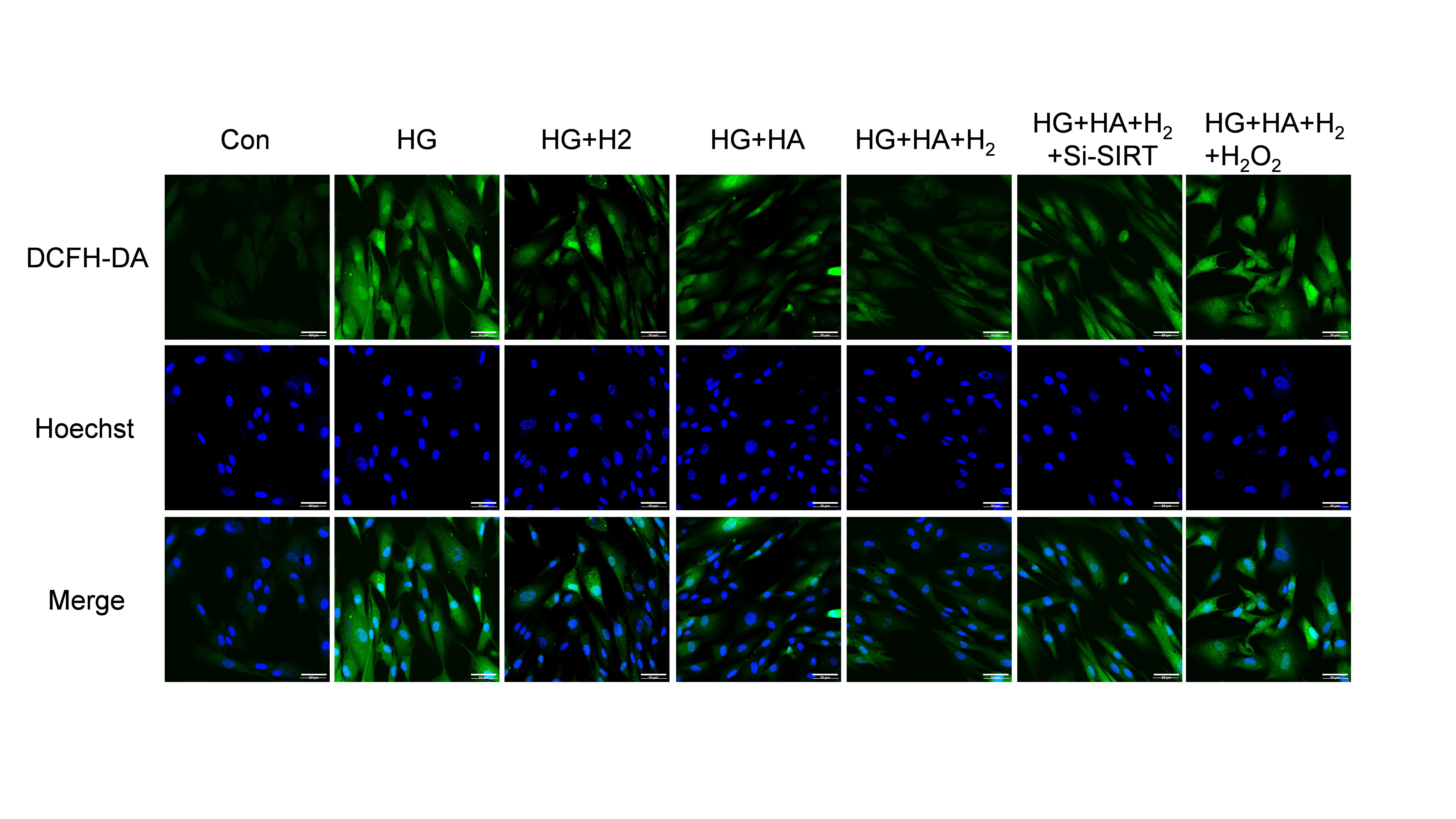


A


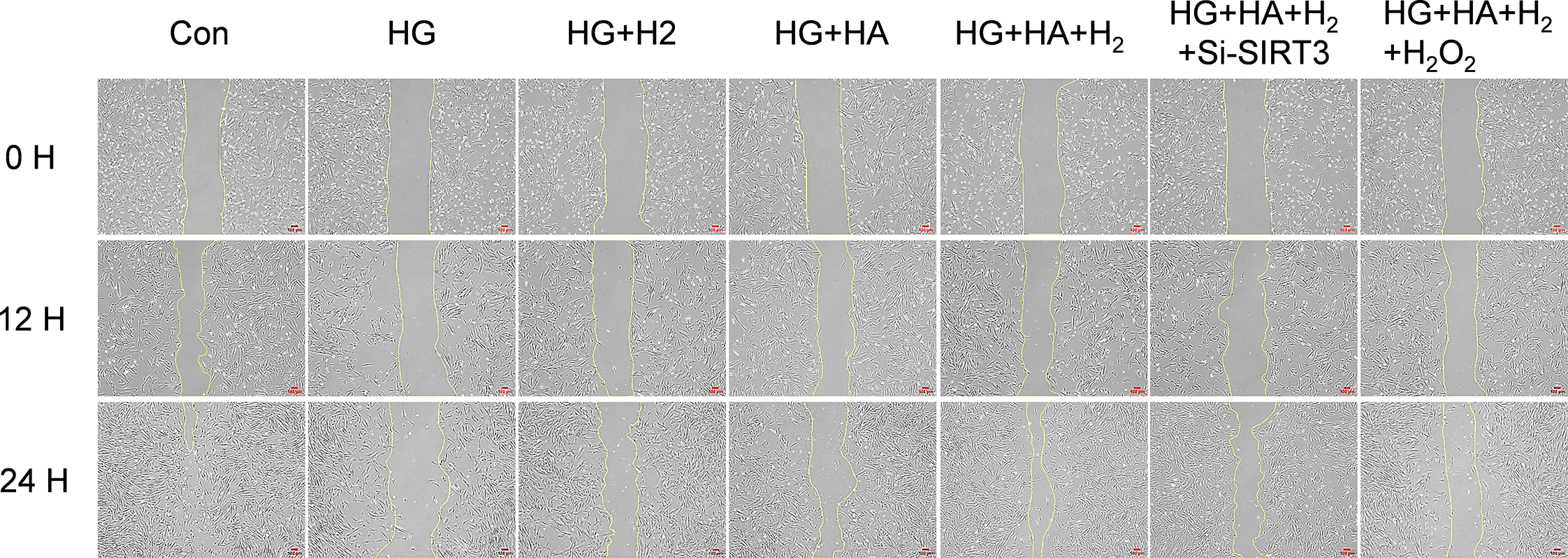


C

B

D


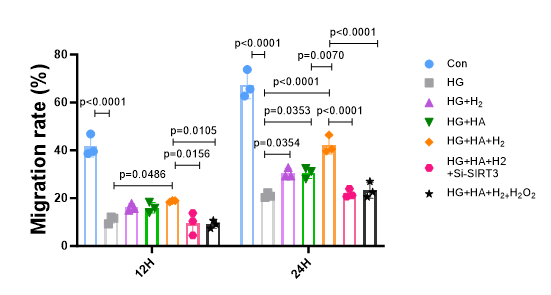

Supplement: Supplementary file 1 — Figure S1: (A) Digital images of diabetic wounds. (B) Corresponding wound size statistic (n = 6). Figure S2: (A) CCK8 assay to assess cell viability in cultures with different glucose DMEM (n = 4). (B) CCK‐8 assay to evaluate the effect of 75 mM high‐glucose‐induced osmotic pressure on cell viability (n = 6). Figure S3: (A) Cellular morphology observed using an optical microscope. (B) CCK8 assay to evaluate cell viability under different culture conditions (n = 5). Figure S4: (A) Western blot results showing the protein levels of SIRT3 in HFF‐1 cells treated with hyaluronic acid and hydrogen in the high‐glucose environment (n = 6). (B) Quantified data showing the protein expression of (A). Figure S5: (A) JC‐1 signal in HFF‐1 cells was examined by fluorescence confocal microscopy. Cells were labeled with Hoechst to show the nucleus (blue) and stained with JC‐1 to show the mitochondria. Double staining of cells with JC‐1 is shown: green for monomers, red for aggregates (scale bar = 50 μm, n = 3). (B) Mean optical density of the ratio of aggregate to monomer (n = 3). Figure S6: (A–G) Western Blot was used to detect SIRT3, FOXO3A, PINK1, PARKIN, P62 and LC3 II/I. The bands represent protein levels (n = 3). Figure S7: (A) Representative images of tube formation by HUVECs cultured on Matrigel for 6 h under different treatments. (B) number of branch points. (C) number of meshes. (D) number of nodes and (E) total tube length per field (scale bar = 100 μm, n = 3). Figure S8: (A, B) The generation of intracellular ROS production was tested by DCFH‐DA probe‐based immunofluorescence staining (scale bar = 50 μm, n = 3). (C, D) The effect of the synergistic effects of hyaluronic acid and hydrogen on the migration of HFF‐1 cells in a high‐glucose environment was detected by scratch wound assays (scale bar = 100 μm, n = 3). [file JDB-18-e70209-s001.doc]
