# Supplementary material for: Hydrogen‐Enriched Hyaluronic Acid Dressing Ameliorates Diabetic Foot Ulcer via Promoting Mitophagy
Source: J Diabetes. 2026 Mar 29;18(4):e70209. doi: 10.1111/1753-0407.70209 (PMC13112070; doi:10.1111/1753-0407.70209)
Supplement: Supplementary file 2 — Table S1: Inclusion criteria for clinical samples. Table S2: Comparison of general data between the two groups. Table S3: Analysis of covariance for treatment effect on wound healing rate after adjusting for baseline characteristics. [file JDB-18-e70209-s002.doc]

**Supplemental Table**

Table S1 Inclusion Criteria for Clinical Samples

| Diagnostic Criteria | Patients diagnosed with diabetes mellitus (DM) or diabetic foot ulcer (DFU) |
| --- | --- |
| Age Range | Adult population (18-75 years old), regardless of gender |
| Ulcer Grading | DFU assessed as grade 2-4 according to the Wagner grading standard |
| Others | 1.No history of malignant tumor, autoimmune disease, severe infection, etc. in the past 6 months;  2. No coagulation disorder, tissue hemostasis can be achieved after debridement, and VSD treatment can be tolerated;  3. Not participating in other clinical trials at the same time.  4. Voluntarily participate in this study and sign the informed consent form. |

Table S2 Comparison of general data between the two groups

| Group | Gender | Age | DFU duration | HbA_1c_ level | Wagner grade | | |
| --- | --- | --- | --- | --- | --- | --- | --- |
|  | (Male/Female) | (mean±SD, years) | (mean±SD, days) | (mean±SD, %) | Ⅱ | Ⅲ | Ⅳ |
| VSD group | 16/14 | 64.73±9.99 | 32.93±11.57 | 8.34±1.01 | 6 | 20 | 4 |
| VSD+H_2_ group | 17/13 | 66.17±12.10 | 29.30±12.67 | 8.37±0.99 | 8 | 16 | 6 |
| X^2^ | 0.067 | — | — | — | 1.130 | | |
| T value | — | -0.500 | 1.160 | -0.141 | — | | |
| P value | 0.795 | 0.619 | 0.251 | 00888 | 0.568 | | |

Table S3. Analysis of Covariance for Treatment Effect on Wound Healing Rate After Adjusting for Baseline Characteristics

| **Source of Variance** | df | F | P-Value | Partial η² |
| --- | --- | --- | --- | --- |
| **Corrected Model** | 6 | 10.41 | **< 0.001** | 0.536 |
| **Treatment Group** | 1 | 52.07 | **< 0.001** | 0.491 |
| **Initial Wound Area** | 1 | 0.07 | 0.788 | 0.001 |
| **Age** | 1 | 0.03 | 0.872 | 0.001 |
| **Gender** | 1 | 0.71 | 0.402 | 0.013 |
| **Diabetes Duration** | 1 | 0.08 | 0.784 | 0.001 |
| **HbA1c** | 1 | 0.31 | 0.577 | 0.006 |

**Adjusted Healing Rate, % (95% CI)**

| Group | Adjusted Healing Rate, % (95% CI)¹ | vs. VSD group (Adjusted Difference, % [95% CI] ) | P-Value |
| --- | --- | --- | --- |
| **VSD group** | 10.69±0.68 | — | — |
| **VSD+H_2_ group** | 12.79±0.68 | **2.10±0.59** | **< 0.001** |
